# Tuberculosis and HIV co-infection in Congolese children: risk factors of death

**DOI:** 10.11604/pamj.2019.33.326.18911

**Published:** 2019-08-27

**Authors:** Olivier Mukuku, Augustin Mulangu Mutombo, Christian Ngama Kakisingi, Jacques Mbaz Musung, Stanislas Okitotsho Wembonyama, Oscar Numbi Luboya

**Affiliations:** 1 Department of Research, Institut Supérieur des Techniques Médicales, Lubumbashi, Democratic Republic of Congo; 2 Department of Pediatrics, University of Lubumbashi, Lubumbashi, Democratic Republic of Congo; 3 Department of Internal Medicine, University of Lubumbashi, Lubumbashi, Democratic Republic of Congo; 4 Department of Public Health, University of Lubumbashi, Lubumbashi, Democratic Republic of Congo

**Keywords:** Tuberculosis, HIV, Co-infection, Child, Mortality, Lubumbashi

## Abstract

**Introduction:**

Human immunodeficiency virus (HIV) and tuberculosis (TB) are the leading causes of death from infectious disease worldwide. The prevalence of HIV among children with TB in moderate to high prevalence countries ranges between 10% and 60%. This study aimed to determine the prevalence of HIV infection among children treated for TB in Directly Observed Treatment Short-Course (DOTS) clinics in Lubumbashi and to identify risk of death during this co-infection.

**Methods:**

This is a cross-sectional study of children under-15, treated for tuberculosis from January 1, 2013 to December 31, 2015. Clinical, paraclinical and outcome data were collected in 22 DOTS of Lubumbashi. A statistical comparison was made between dead and survived HIV-infected TB children. We performed the multivariate analyzes and the significance level set at p-value <0.05.

**Results:**

A total of 840 children with TB were included. The prevalence of HIV infection was 20.95% (95% CI: 18.34-23.83%). The mortality rate was higher for HIV-infected children (47.73%) compared to HIV-uninfected children (17.02%) (p<0.00001). Age <5 years (aOR=6.50 [1.96-21.50]), a poor nutritional status (aOR=23.55 [8.20-67.64]), and a negative acid-fast bacilli testing (aOR=4.51 [1.08-18.70]) were associated with death during anti-TB treatment.

**Conclusion:**

TB and HIV co-infection is a reality in pediatric settings in Lubumbashi. High mortality highlights the importance of early management.

## Introduction

The most important chronic diseases affecting children in sub-Saharan Africa are tuberculosis (TB), Human Immunodeficiency Virus (HIV) infection and malnutrition. These three diseases often have similar clinical aspects or are frequently associated with the same patient. Globally, the epidemic of HIV infection has been accompanied by a serious epidemic of TB [1,2]. HIV infection is the main risk factor for tuberculosis because HIV promotes the progression of latent or recent infections of Mycobacterium tuberculosis into active disease and also increases the frequency of TB [2,3]. Both pathologies are the two leading causes of infectious disease deaths worldwide [4]. There are well-established epidemiological and biological synergies between HIV and TB, which influence the distribution, progression and outcomes of both infections. The HIV epidemic is a key factor in the resurgence of TB incidence worldwide and HIV is the pre-incident risk factor for TB development [2,4]. According to the latest global estimates, 36.9 million people are living with HIV, 70% of whom live in sub-Saharan Africa [5] and 1.7 billion people are affected by latent TB [6,7]. In 2017, UNAIDS estimated 1.8 million children under-15 living with HIV [5]. World Health Organization (WHO) estimated that 10% (1 million) of the 10.4 million new TB cases recorded in 2016 were children and 11% (1.2 million) of those with TB were HIV positive [6]. But specifically for children, there is limited data on the incidence of HIV infection among children with TB, and the information available is difficult to interpret because of problems with diagnosis, under-reporting and selection of the studied populations (hospitals rather than community) [4]. WHO estimates that HIV prevalence among children with TB in medium and high prevalence countries is between 10% and 60% [8,9] and varies with background rates of HIV infection [10].

HIV-TB co-infection has been well explored in adults in sub-Saharan Africa, but has so far been insufficiently studied in children because of the difficulty of establishing an accurate diagnosis of TB in children in particular by microscopy. It is considered a major public health problem, particularly in resource constrained settings like Democratic Republic of Congo (DRC). The DRC is among the 30 countries heavily affected by TB; it ranks eleventh in the world and third in Africa [6]. In 2016, estimates report 32,000 cases of all forms of TB in children under-15 [6]. HIV infection and tuberculosis are two major burdens whose control or elimination is a huge challenge for African states and their health services. The co-infection tuberculosis and HIV in children is distinguished by its particular aspects. Epidemiologically, it is a direct consequence of adult tuberculosis and reflects the transmission of the disease in the community. The often atypical clinical manifestations (extrapulmonary tuberculosis) and the bacteriological and anatomo-pathological diagnosis remain difficult [11,12].

In the DRC, the fight against TB is the task entrusted to the National Tuberculosis Program (NTP), it is part of the direction of the fight against the diseases. In order to achieve its various objectives, the NTP recommends an integrated approach to TB control activities in Primary Health Care (PHC) structures in line with the strategy for strengthening the health system. The structure that provides diagnosis and treatment for tuberculosis patients is called the Directly Observed Treatment Short-Course (DOTS) clinics. The DOTS is the functional unit of the NTP [13]. The objectives of this study are to determine the prevalence of HIV infection among all children treated for TB in DOTS clinics in Lubumbashi (DRC) and to identify risk of death during this co-infection.

## Methods

We conducted a cross-sectional study. Our team collected data from the 22 DOTS in Lubumbashi, the capital of the Haut-Katanga Province in the DRC. The target patients were children under-15 admitted to the DOTS with a diagnosis of tuberculosis from January 1^st^, 2013 to December 31^th^, 2015. Of a total of 8699 TB patients in this period, 840 were under 15 years old. The children were classified into two groups according to their HIV status. A group of HIV-positive TB children and a group of HIV-negative TB children have been formed.

The HIV testing strategy was that proposed by national standards and guidelines [14]. This strategy recommends the use of two rapid tests namely, Determine™ HIV1/2 and Unigold™ HIV. In the event of a discrepancy in rapid tests, HIV infection was confirmed by an Enzyme-Linked Immunisorbent Assay (ELISA). For children under 18 months of age, the diagnosis of HIV infection was made by Polymerase Chain Reaction (PCR). The diagnosis of smear-positive pulmonary TB (PTB) was made if a patient met at least one of the following criteria according to the standard definitions of the NTP of DRC adopted from WHO [13,15,16]: Acid-fast bacilli (AFB) in at least two sputum samples, The presence of AFB in a sputum sample and X-ray compatible with active pulmonary TB or AFB in sputum specimen and positive sputum culture for Mycobacterium tuberculosis.

The diagnosis of smear-negative PTB was made if the patient met the following criteria [13,15,16]: radiographic signs compatible with active PTB, AFB negative in three sputum samples, Decision to treat the patient as PTB by the medical staff of the hospital where the patient was admitted, Ineffectiveness of non-specific antibiotic therapy and.

Diagnosis of extrapulmonary tuberculosis (EPTB) was based on fine needle aspiration cytology or biochemical analyses of cerebrospinal/pleural/ascitic fluid or histopathological examination or strong clinical evidence consistent with active EPTB, followed by a decision of a clinician to treat with a full course of antituberculosis chemotherapy [13,15,16]. We also included patients who were unable to produce appropriate sputum specimens but whose symptoms and clinical history were consistent with active TB. Patients with both PTB and EPTB have been classified as PTB in accordance with the definition of the WHO [15].

Demographic and clinical information was collected from medical records using a standardized data collection form. The following data were extracted from the records of each patient: age, sex, contagion with a known TB case, history of TB, presence or absence of BCG vaccine scar, and time to onset of symptoms before admission to the service. The following general and physical clinical signs were systematically recorded: asthenia, weight loss, anorexia, unexplained fever (>2 weeks) greater than or equal to 38°C, persistent cough (>2 weeks), weight-for-age z-score (WAZ) ≤-2 SD and peripheral lymphadenopathy. The results of the direct tuberculosis bacillus test, as well as those of the hemogram and HIV serology of each patient at admission were also recorded. AFB was performed on sputum or gastric tube fluid from patients with pulmonary or mixed forms of tuberculosis on admission: a direct examination by the Zielh-Neelsen technique and culture of mycobacteria on the Löwenstein-Jensen medium.

The results of tuberculosis treatment have been classified into six categories according to WHO definitions: cured, completed treatment, died, defaulted treatment, failure and transfer-out [15]. Children who were cured and those who had completed treatment were classified as having successful treatment (or having a favorable outcome) [13,15,17]. All information collected was analyzed using STATA 12 software. For each factor studied, the proportions were compared between groups of HIV-positive and HIV-negative TB children and between dead and survived HIV-infected TB children. Chi square test was used for comparison of percentages in univariate and multivariate analysis. The significance level of all observed differences was set at p<0.05.

This study was authorized by the medical ethic committee of the University of Lubumbashi and the Health authority of Haut-Katanga Province before data collection. Patient records/information was anonymized and de-identified prior to analysis to ensure confidentiality of individual patient information.

## Results

A total of 840 children with TB were included, of whom 176 were HIV-infected, an HIV infection of 20.95% (95% CI: 18.34-23.83%). Table 1 presents the epidemiological data, clinics and laboratory investigations of the 840 TB children according to their HIV status. The history of anti-TB treatment, prolonged fever, and EPTB were significantly more common in HIV-infected children than in HIV-uninfected children (p<0.05). The positive TST was significantly lower in HIV-infected children than in HIV-uninfected children (56.82 vs 65.21%) (p=0.0399). The same is true for nutritional status, we noted that 45.45% of HIV-infected children had a weight-for-age z-score ≤-2 SD against 18.83% in HIV-uninfected children (p<0.00001). The anti-TB treatment outcomes of HIV-infected children included in this study were 75 (42.61%) favorable, 8 (4.55%) defaulted, 84 (47.73%) died, 5 (2.84%) failure, and 4 (2.27%) transferred out. The mortality rate was higher for HIV-infected children (47.73%, n=84) compared to HIV-uninfected children (17.02%, n=113) (p <0.00001). The frequency of favorable anti-TB treatment outcomes was higher for HIV-negative patients (76.50%, n=508) compared to HIV-positive patients (42.61%, n=75) (Table 2). Table 3 shows the risk factors for death in HIV-infected children. Statistical analysis showed that age ≤5 years (aOR=6.50 [1.96-21.50]), a z-score weight-for-age ≤-2 SD (aOR=23.55 [8.20-67.64]), and a negative acid-fast bacilli testing (aOR=4.51 [1.08-18.70]) were most closely associated with mortality during anti-TB treatment.

**Table 1:** Participants characteristics

Variable	Total, n (%) (N=840)	HIV-infected, n (%) (n=176)	HIV-uninfected, n (%) (n=664)	p
Age ≤5 years	202 (24.05)	46 (26.14)	156 (23.49)	0.5286
Mean age ± SD (years)	8.27±3.95	8.30±3.92	8.16±4.08	0.6767
Male sex	438 (52.14)	89 (50.57)	349 (52.56)	0.6998
Absence of BGC vaccine scar	120 (14.29)	22 (12.50)	98 (14.76)	0.5219
History of contact with a smear-positive TB patient	595 (70.83)	128 (72.73)	467 (70.33)	0.5971
Re-treatment	57 (6.79)	36 (20.45)	21 (3.16)	<0.00001
Fever ≥2 weeks	615 (73.21)	140 (79.55)	475 (71.54)	0.0415
Cough ≥2 weeks	463 (55.12)	99 (56.25)	364 (54.82)	0.7994
Lymphadenopathy	380 (45.24)	79 (44.89)	301 (45.33)	0.9838
Anorexia	580 (69.05)	124 (70.45)	456 (68.67)	0.7170
Weight loss	547 (65.12)	123 (69.89)	424 (63.86)	0.1604
Night sweats	376 (44.76)	72 (40.91)	304 (45.78)	0.2842
Poor nutritional status (WAZ ≤ -2 SD)	205 (24.40)	80 (45.45)	125 (18.83)	<0.00001
EPTB	123 (14.64)	37 (21.02)	86 (12.95)	0.0100
TST positive	533 (63.45)	100 (56.82)	433 (65.21)	0.0399
AFB positive	201 (28.03)	34 (24.46)	167 (28.89)	0.3475

**HIV**: human immunodeficiency virus; **SD**: standard deviation; **TB**: tuberculosis; **TST**: Tuberculin skin test; **AFB**: Acid Fast Bacilli; **WAZ**: Weight-for-Age Z-score; **EPTB**: Extra pulmonary tuberculosis

**Table 2:** Anti-TB treatment outcomes among 840 TB children in Lubumbashi

Anti-TB treatment outcomes	Total, n (%) (N=840)	HIV-infected, n (%) (n=176)	HIV-uninfected, n (%) (n=664)	p
Successful treatment *	583 (69.40)	75 (42.61)	508 (76.50)	
Died	197 (23.45)	84 (47.73)	113 (17.02)	<0.00001
Failure	15 (1.79)	5 (2.84)	10 (1.51)	
Defaulted	32 (3.81)	8 (4.55)	24 (3.61)	
Transferred out	13 (1.55)	4 (2.27)	9 (1.36)	

* Cured + Completed treatment

**Table 3:** Risk factors for death in HIV infected children

Variable	Total (N=159)	Died, n (%) (n=84)	Survived, n (%) (n=75)	Crude OR [95% CI]	Adjusted OR [95% CI]
**Age ≤5 years**					
Yes	36	27 (75.00)	9 (25.00)	3.47 [1.51-7.99]	6.50 [1.96-21.50]
No	123	57 (46.34)	66 (53.66)	1.00	1.00
**Sex**					
Male	81	43 (53.09)	38 (46.91)	1.02 [0.54-1.90]	0.98 [0.41-2.31]
Female	78	41 (52.56)	37 (47.44)	1.00	1.00
**Re-treatment**					
Yes	34	22 (64.71)	12 (35.29)	1.86 [0.85-4.08]	1.27 [0.37-4.39]
No	125	62 (49.60)	63 (50.40)	1.00	1.00
**History of TB contact**					
Yes	116	62 (53.45)	54 (46.55)	1.09 [0.54-2.20]	1.32 [0.49-3.54]
No	43	22 (51.16)	21 (48.84)	1.00	1.00
**BGC vaccine scar**					
No	20	12 (60.00)	8 (40.00)	1.39 [0.53-3.62]	1.84 [0.45-7.42]
Yes	139	72 (51.80)	67 (48.20)	1.00	1.00
**Anorexia**					
Yes	113	62 (54.87)	51 (45.13)	1.32 [0.66-2.63]	1.04 [0.38-2.82]
No	46	22 (47.83)	24 (52.17)	1.00	1.00
**Night sweats**					
Yes	62	28 (45.16)	34 (54.84)	0.60 [0.32-1.14]	0.88 [0.34-2.28]
No	97	56 (57.73)	41 (42.27)	1.00	1.00
**Weight loss**					
Yes	112	61 (54.46)	51 (45.54)	1.25 [0.63-2.47]	1.42 [0.51-3.92]
No	47	23 (48.94)	24 (51.06)	1.00	1.00
**Poor nutritional status (WAZ ≤ -2 SD)**					
Yes	73	60 (82.19)	13 (17.81)	11.92 [5.56-25.56]	23.55 [8.20-67.64]
No	86	24 (27.91)	62 (72.09)	1.00	1.00
**Fever >2 weeks**					
Yes	130	67 (51.54)	63 (48.46)	0.75 [0.33-1.69]	0.69 [0.23-2.06]
No	29	17 (58.62)	12 (41.38)	1.00	1.00
**Cough >2 weeks**					
Yes	87	38 (43.68)	49 (56.32)	1.00	1.00
No	72	46 (63.89)	26 (36.11)	2.28 [1.20-4.33]	0.53 [0.19-1.47]
**Lymphadenopathy**					
Yes	71	39 (54.93)	32 (45.07)	1.16 [0.62-2.18]	1.37 [0.54-3.45]
No	88	45 (51.14)	43 (48.86)	1.00	1.00
**Type of TB**					
EPTB	36	23 (63.89)	13 (36.11)	1.79 [0.83-3.87]	1.42 [0.35-4.76]
PTB	123	61 (49.59)	62 (50.41)	1.00	1.00
**TST**					
Negative	68	38 (55.88)	30 (44.12)	1.24 [0.66-2.32]	0.39 [0.15-1.01]
Positive	91	46 (50.55)	45 (49.45)	1.00	1.00
**AFB testing**					
Positive	30	9 (30.00)	21 (70.00)	1.00	1.00
Negative	129	75 (58.14)	54 (41.86)	3.24 [1.37-7.62]	4.51 [1.08-18.70]

**HIV**: human immunodeficiency virus; SD: standard deviation; **TB**: tuberculosis; **TST**: Tuberculin skin test; **AFB**: Acid Fast Bacilli; **WAZ**: Weight-for-Age Z-score; **EPTB**: Extra pulmonary tuberculosis; **PTB**: Pulmonary tuberculosis; **OR**: odds ratio; 95% CI: 95% confidence interval

## Discussion

This study found that the prevalence of HIV-TB co-infection among TB patients was 20.95%, which is higher as compared with 11.8% prevalence that was reported in Abidjan (Côte-d'Ivoire) by Sassan-Murokro *et al*. [18]. This rate is lower than the 25.7% found in Bangui (Central African Republic) by Bobossi-Serengbe *et al*. [19], than the 35% recorded in Brazzaville (Republic of Congo) by Loufoua-Lemay *et al*. [20]. In South Africa, Madhi *et al*. [21] reported 42% HIV infection in all children treated for tuberculosis in Johannesburg and, Jeena *et al*. [22] found only 48% of those whose tuberculosis was confirmed bacteriologically in Durban. Estimated rates of HIV infection among TB children vary widely around the world, in part depending on whether the study is conducted in an area where HIV infection is endemic or not. This study showed the increased mortality risk for HIV-infected TB children as compared to HIV-uninfected TB children. A possible explanation is the higher clinical severity of disease at the admission [23].

This study found that HIV-infected children were more likely to have extrapulmonary forms that could also explain this high mortality in these children. This high mortality in HIV-infected children could be attributed to late diagnosis and delayed antiretroviral treatment (ART). In sub-Saharan Africa, HIV infection in the pediatric population is often diagnosed very late and especially in the course of the disease, and when ART starts late, the child´s immune system may already be seriously compromised [24]. Early pediatric HIV testing is not done because some parents / guardians rely only on their child’s apparent good health, and others believe that children infected during the perinatal period do not survive until the end of their childhood [24,25]. Nearly half of HIV-infected TB children died during anti-TB treatment. HIV-TB co-infection was significantly associated with death and this is consistent with previous studies elsewhere [26-28]. Lolekha found that HIV-infected TB children were 6.9 times more likely to die than HIV-negative children [29]. HIV contributes substantially to the burden of childhood TB. HIV-TB co-infection is frequently associated with multiple infections complicating diagnosis and treatment, leading to increased morbidity and mortality [26]. Biruk *et al*. [30] explains that this would be related to pill burden, increase in adverse effect, drug-to-drug interaction, and immune reconstitution inflammatory syndrome.

In this study, a number of factors associated with death in HIV-infected TB children have been reported. The mortality rate among children under-5 was significantly higher than those over 5 years old. Our results corroborate those of studies elsewhere [26,31]. In line with this finding, a study in Malawi found a decline in death rates with advanced age [32]. Younger children, especially those younger than two years of age, are at increased risk of death from infectious diseases, including tuberculosis due to the immaturity of the immune system. In addition, these young children are frequently affected by disseminated tuberculosis and tuberculous meningitis, which are associated with high mortality [12,33,34].

In this observation, the characteristics of HIV-infected TB children associated with death during anti-TB treatment were being negative AFB testing. Previous studies reported that TB patients with unsuccessful treatment outcome were being smeared negative pulmonary TB [35-39]. This could be due to the treatment outcome monitoring of smear-positive TB patients who, apart from clinical progression, have repeated sputum tests [30]. Contrary to our findings, Bloss *et al*. [40] and Hailu *et al*. [26] found that children with smear-positive PTB died more than those with smear-negative smears and assume that it is related to late-stage TB in the majority of patients in their series.

Our study showed that 82.19% of the dead children had a poor nutritional status. We observed a significant correlation between nutritional deficit and mortality in HIV-infected TB children. A nutritional deficit and a lower concentration of serum albumin have been found to be important risk factors for mortality in TB patients in previous studies [41,42]. According to Kim, malnutrition would be a clinical finding reflecting the severity of TB and the determinant of mortality would be the severity of TB rather than malnutrition itself [42].

### Limitations of the Study

Therefore, this study is not without limits and the results of this study should be interpreted with the following limitations. The major limitation of this work is the use of retrospective data, which is totally restricted to what is documented in the TB registers. Predictor variables which might affect outcome like treatment adherence level, comorbidities, modalities of HIV care and ART usage, other opportunistic infections as well as behavioral and social factors, were not available in the TB national system.

## Conclusion

High proportion of death in HIV-infected TB children was documented in this study. Moreover, the following risk factors were identified as predictors of death: age ≤ 5 years, poor nutritional status, and negative AFB testing. Based on the findings of this study, we recommend that emphasis has to be given for children with high risk of death and targeted interventions should be carried out.

### What is known about this topic

- In the DRC, infectious disease are a major public health problem with an important rate of morbidity and mortality among children;
- Mortality due to TB and HIV on young children is separately higher.

### What this study adds

- In this study, the incidence of HIV among children with TB is 20.95% and children with HIV-TB coinfection had a higher mortality rate as compared to the HIV uninfected children with TB;
- The proposed study is the first study in our setting, integrating a multivariate analysis to identify the risk factors of death in HIV-infected TB children in Lubumbashi, Democratic Republic Congo;
- In our environment, the risk factors of death for TB- and HIV co-infection on children are: age ≤5 years, poor nutritional status, and negative AFB testing.

## Competing interests

The authors declare no competing interests.
